# Supplementary material for: Sources of diagnostic delay for people with Crohn’s disease and ulcerative colitis: Qualitative research study
Source: PLoS One. 2024 Jun 10;19(6):e0301672. doi: 10.1371/journal.pone.0301672 (PMC11164383; doi:10.1371/journal.pone.0301672)
Supplement: S1 File — (DOCX) [file pone.0301672.s001.docx]

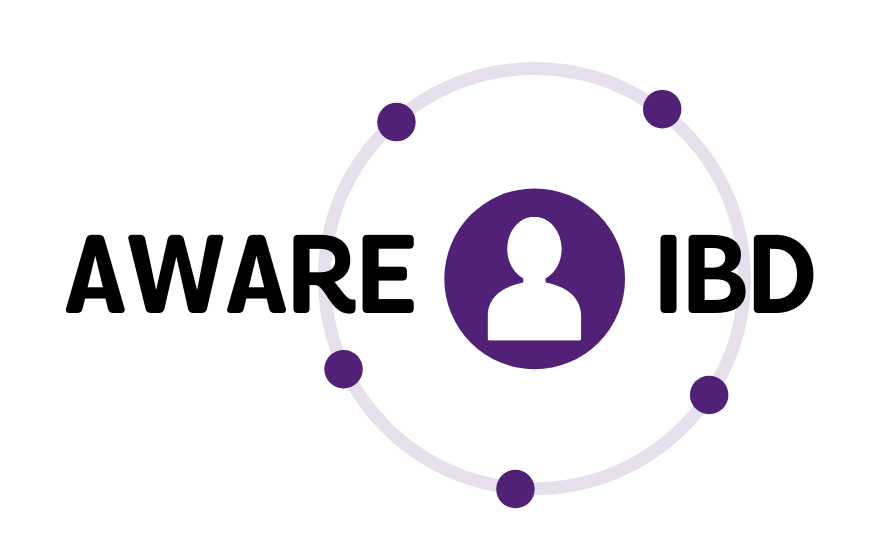


**Patient Experiences of the Journey to Diagnosis Sub-Study – Interview Guide**

**Introduction**

*The following interview guide is not intended to be used as a script for interviewers to follow strictly, in full detail.*

*The first-level bullet points in each section are core questions to be asked but much of the detail beneath each first-level question consists of further prompts for the interviewer – a checklist of potential follow-up questions they may ask depending on the nature and level of detail in the interviewee’s response to the initial question.*

*Interviewees will also have had varying experiences in their journeys to diagnosis and some journeys will be longer or shorter, or less or more complex, according to circumstances. Not all questions, or levels of detail will apply to all interviewees.*

*Many of the listed questions will, therefore, not need to be asked, depending on the details of each interviewee’s experiences and their responses to initial questions.*

*(Footnotes are also only prompts for interviewers.)*

1. ***Detecting physical changes (symptoms, onset, impact, severity)***

*This first part is about before you were diagnosed, and before you first saw any healthcare professionals about any symptoms*

- What physical symptoms first concerned you?
  - What were the first indications that something was different or ‘not right’?
  - Did you experience any changes in your bowel habits?
- When did these symptoms begin? *(date or month/year)*
- As well as any such symptoms, did you feel, see or smell anything different or unusual?
- Would you say that before this you were previously aware of what healthy bowel movements/stool would typically be like?
- Did these symptoms impact aspects of your day-to-day life? How?
  - E.g. on your career, education, relationships, mood, mental health or well-being, or sense of self?
  - Did your symptoms cause you to stop doing anything you would normally do?
- Did you use any ways of keeping track of your symptoms or experiences either before or after you first consulted a healthcare professional?
- Did your symptoms represent a significant change compared to your normal state of health or was there a slow creep of symptoms, or slowly worsening symptoms, that only caused concern once they reach a certain level?
- Did you take any over-the-counter medications to try to deal with your symptoms?
  - Can you recall what?
  - When was this? *(date or month/year)*
  - Did this help at all? How and how long for?
- Had you heard of Crohn’s Disease or Ulcerative Colitis (or just Colitis) before you first experienced symptoms?
- If, no, when did you first hear about them? Did you learn about them during your journey to diagnosis or only when diagnosed?
- Did anyone suggest you might have Crohn’s or colitis before your diagnosis? Did anyone suggest it may be some other problem or condition?
- Did you think you might have Crohn’s or colitis, before your diagnosis? What made you think this?
- Do you have any history of Inflammatory Bowel Disease in your family?
- What would you describe as the worst points before you were diagnosed? Did these have any effect on your decision to seek help (or to not seek help, or put off seeking help)?

1. ***Perceiving reasons to discuss symptoms with healthcare professionals***

*This next part is about when and why you decided to seek some medical help about your symptoms*

- What made you seek help from a healthcare professional?
- When did you *decide* to take action to consult someone about your symptoms? *(date or month/year) (NB not when consultation happened, but when they decided to take action)*
  - How long was this after your first symptoms appeared?
  - Did you see a healthcare professional when you first had symptoms or was your first consultation at a later point?
  - If later, what was the immediate trigger for seeing a healthcare professional at that time?
  - Was there one thing (event, symptom) that made you decide to see someone or was it a gradual build-up of symptoms?
- Was there anything that stopped you from seeking help at any point?
  - Such as:
  - getting time off work
  - caring responsibilities for dependents
  - travel restrictions
  - limited or difficult access to healthcare professionals
  - feeling unable to discuss symptoms (e.g. with family and friends)
  - family or friends dismissed your symptoms as not serious
  - any religious or cultural concerns
  - anxieties or fears that it may be something more serious
  - anything else?
  - (If interviewee’s symptoms and consultations were since March 2020, prompt them as to whether Covid caused any reluctance or inability to seek help.)
- Would anything have made you feel more comfortable or made it easier for you to seek help at any point?

1. ***Consultations with healthcare professionals***

*This part is about who you saw and what happened when you did seek medical help for your symptoms*

- When did you first seek help for your symptoms? *(date or month/year)*
- Who did you consult? (NHS? Private consultation? GP, practice nurse, hospital appointment, community pharmacist, etc.)^[[1]](#footnote-1)^
- Did you feel comfortable discussing your symptoms with the healthcare professional?
  - Did you discuss all your symptoms with the healthcare professional?
  - Were there any symptoms you were uncomfortable discussing, did not discuss or only partly discussed?
  - Were there any words, terms or language you felt uncomfortable using or unable to use in describing or discussing your symptoms?
- Were the healthcare professionals you consulted supportive and understanding of your concerns and symptoms?
  - Did you feel your symptoms were taken seriously by the healthcare professional?
  - Did you feel you were being properly listened to?
  - Did you need to talk to more than one healthcare professional before you or your symptoms were taken seriously?
  - If so, who and when? *(date or month/year)*
- Did you feel you were asked enough questions (for enough detail) about your symptoms? Can you recall what question you were asked?
  - Did any particular questions you remember being asked seem especially important?
  - Were you asked if you had a family history of such symptoms?
  - Were there any questions you expected to be asked that were not asked?
  - How did you feel about your initial consultation(s) overall?
- Did the first person you consulted conduct, or order, any tests (e.g. blood tests or other investigations) or prescribe any medication?
  - At what stage were any tests done (i.e. after seeing which healthcare professional)?
  - When were different tests/investigations done? *(date or month/year)*
  - What tests or investigations were conducted^[[2]](#footnote-2)^
    - By the first person you saw
    - By someone you were referred to by them
- How did the need for tests and the tests themselves make you feel?
  - Were your reassured or concerned (or both)?
  - How did you feel while waiting for a test or investigation?
- How long did you have to wait once tests were ordered, for them to take place? And how long to get results? *(try to get dates or month/year)*
- How did you feel about the time taken to get tests or investigations scheduled, and then the time taken to get the results?
- Did the first person you consulted refer you on to another healthcare professional or other service?
  - Was that referral immediately upon your first consultation or some time later? When?
  - How many times (over what time period) did you see that first person before being referred on?
  - How long was it between referral and seeing the person you were referred to?
- Did you consult different health professionals, independently? ^[[3]](#footnote-3)^ (I.e. not including referrals)
  - If so, at what stage, and why? When was this? *(date or month/year)*
- Did you attend a hospital’s A&E department at any time, related to these symptoms?
  - If so, when? *(date or month/year)*
  - Were any tests done? What?
  - Were you given any treatment? What?
  - Were you admitted as an in-patient? What happened as a result?
  - Were you referred by A&E to another healthcare professional or service / given an appointment for any such follow-up?
- Were your symptoms developing during the time you were having different consultations?
  - If so, how?
  - Were you able to update the person you had seen about changes or developments in your symptoms?
- Were you diagnosed with any other conditions before Crohn’s or colitis, as a result of your symptoms and/or any investigations or tests? If so, when? *(date or month/year)*
- Did you receive any treatments for your symptoms, before your Crohn’s or colitis was formally diagnosed? If so, when? *(date or month/year)*
- Did you decline any tests or investigations at any point? Why?

1. ***Diagnosis***

*This part is about when you received a formal diagnosis of Crohn’s or colitis*

- Who made your diagnosis and told you what it was? When was this? *(date or month/year)*
  - Was it a hospital consultant/specialist, IBD nurse, GP, endoscopist, surgeon, etc.
- Do you know what finally confirmed your diagnosis?
  - Particular test result?
  - Case notes review?
- Were you given any further information about your diagnosis?
  - E.g. an information pack or any leaflets
  - Were you told about any support organisations such as Crohn’s & Colitis UK or others? (Which ones?)
- How did you feel at the point of diagnosis? What were your first thoughts?
- Did you feel the way you were told about your condition was done sensitively?
- Did you feel able to tell others (family, friends, colleagues, employer) about your diagnosis?
  - How did they react?
- If you were not able to tell others, why was this?
  - You felt you did not need their support?
  - You felt it was too private?
  - You did not feel confident enough to do this?
  - Were you worried about their reaction?
  - What might have helped you in talking to others about your situation?
- Were you immediately placed on a course of treatment of ongoing medication, or scheduled for any surgery, or did more investigations/tests take place first?
  - If so, how long did it take for those to happen? When did they happen? *(date or month/year)*
- Was there any other delay between getting your diagnosis and starting any treatment?
  - What caused this delay?
  - Were further tests/investigations undertaken to determine the best treatment for you?
  - How long did this take; when did this happen? *(date or month/year)*
- Did the first treatment (medication or surgery) help get your IBD under control?
  - How long from diagnosis did it take you to get on a treatment that consistently controlled your IBD?

1. ***Hindsight***

*This is about what might have happened differently, so, looking back on the whole journey to diagnosis from start to finish:*

- What do you know now, that may have been useful to have known earlier?
- Is there anything specific that you or anyone else did that was important in obtaining your diagnosis?
- Is there anything that would have improved your path to diagnosis?
  - Anything that could or should have been done differently by anyone (including you)
- Were there any barriers to getting your diagnosis?
- Do you think your Crohn’s or colitis could have been diagnosed earlier? When? Why?
- What would healthcare professionals have needed to know in order to help you sooner?
- What advice would you give to health care professionals about diagnosing Crohn’s or colitis?
- Is there anything that they could have done to improve your experience of your actual journey to diagnosis?^[[4]](#footnote-4)^

1. ***Summary***

Do you have any other points that you would like to make about your own experience of your journey to diagnosis?

- Is there anything that I have not asked you which you feel it is important for us to know about your journey to diagnosis?
- Do you have any other questions?

1. If more than one of these were consulted then following questions may need repeating for each [↑](#footnote-ref-1)
2. Try to get as much detail as possible. If not forthcoming, or if Faecal Calprotectin not mentioned, prompt to see if they had this test done. [↑](#footnote-ref-2)
3. I.e. did they go elsewhere or see someone else of their own volition rather than as a result of referrals. Also, was it NHS or private? [↑](#footnote-ref-3)
4. Subtle wording point – this is not just ‘how could your diagnosis have happened earlier?’ but also (more so!) ‘how could what actually happened have been a better experience?’ [↑](#footnote-ref-4)
