# Supplementary material for: Sources of diagnostic delay for people with Crohn’s disease and ulcerative colitis: Qualitative research study
Source: PLoS One. 2024 Jun 10;19(6):e0301672. doi: 10.1371/journal.pone.0301672 (PMC11164383; doi:10.1371/journal.pone.0301672)
Supplement: Attachment — Submitted filename: Response to reviewers.docx [file pone.0301672.s002.docx]

**Response to reviewers**

Thank you for the helpful and constructive comments regarding the resubmission of this manuscript. We have incorporated the feedback into the manuscript and feel that the paper is much improved as a result. We will now take each comment in turn and demonstrate how the concerns have been met on a case-by-case basis.

1. Please ensure that your manuscript meets PLOS ONE's style requirements, including those for file naming.

The formatting has been revised according to the style requirements.

2. We note that you have indicated that data from this study are available upon request. PLOS only allows data to be available upon request if there are legal or ethical restrictions on sharing data publicly. For more information on unacceptable data access restrictions, please see http://journals.plos.org/plosone/s/data-availability#loc-unacceptable-data-access-restrictions.

In your revised cover letter, please address the following prompts:

a) If there are ethical or legal restrictions on sharing a de-identified data set, please explain them in detail (e.g., data contain potentially sensitive information, data are owned by a third-party organization, etc.) and who has imposed them (e.g., an ethics committee). Please also provide contact information for a data access committee, ethics committee, or other institutional body to which data requests may be sent.

We have not shared de-identified interview transcripts, as this is concordant with University of Sheffield processes for trial data sets. Furthermore, our participant consent forms and information sheets (which have been approved by Health and Care Research Support Centre, Wales Research Ethics Committee 3) stated that the transcripts would be stored securely for 10 years at the University of Sheffield, and only shared in an anonymised form if considered appropriate by the research team. Therefore, uploading interviews transcripts onto a publicly accessed data repository would oppose what participants agreed to in the informed consent procedure.

Data requests may be sent to ctru@sheffield.ac.uk.

3. Please amend your authorship list in your manuscript file to include author Naseeb Ezaydi.

This manuscript is being submitted under a group authorship ‘AWARE-IBD Diagnostic Delay Working Group’. The members of this group are listed in the acknowledgements section including Naseeb Ezaydi.

4. Please include your full ethics statement in the ‘Methods’ section of your manuscript file. In your statement, please include the full name of the IRB or ethics committee who approved or waived your study, as well as whether or not you obtained informed written or verbal consent. If consent was waived for your study, please include this information in your statement as well.

The methods section has been amended to include the full name of the ethics committee who approved the study and the verbal consent procedure. As shown below:

“The study received ethical approval from the Health and Care Research Support Centre, Wales Research Ethics Committee 3 (16/08/2021). Informed consent was obtained verbally before all interviews. Consent was recorded onto an encrypted dictaphone and also recorded onto a paper consent form, a copy of which was sent to the participant.” **Lines 157-161.**

5. Please review your reference list to ensure that it is complete and correct. If you have cited papers that have been retracted, please include the rationale for doing so in the manuscript text, or remove these references and replace them with relevant current references. Any changes to the reference list should be mentioned in the rebuttal letter that accompanies your revised manuscript. If you need to cite a retracted article, indicate the article’s retracted status in the References list and also include a citation and full reference for the retraction notice.

References 88 – 93 have been removed from the reference list as they are not cited in the main text.

**Reviewer 1 comments:**

Specific remarks:

**Methods:**

How was diagnostic delay precisely defined?

We have added the following to line 104: “For the purpose of this study, our funder defined diagnostic delay to have occurred when a person’s diagnosis by a secondary care specialist took longer than 12 months from first onset of symptoms.”

Were specific pretest applied or excluded in the patients journey, e.g. fecal calprotectin assessment?

This was a naturalistic study in which we asked participants what had happened to them. There was no attempt to prospectively manipulate pathways. Some participants will have received faecal calprotectin assessment; other will not. It was this variation in practice that the study was designed to detect.

Did telephone interviewing as compared to Google Meeting lead to different outcomes/sources ? Why was the cohort approached by two different communication means?

We have added the following at line 165: “To mitigate barriers to inclusion and participation, participants were given a choice of semi-structured interviews conducted by telephone or Google Meet...”

We did not identify any difference in the richness of interviews or the inferences we drew from them that could be related to the medium of their conduct.

**Results:**

Expression of discomfort was present in 7 out of 20 participants ? Or did the 7 not expressing comfort were neutral? Anyhow, how is this number as compared to non-IBD ‘abdominal’ patients?

We are struggling to respond to this comment. Only one variant on the word, “discomfort” appears in our manuscript, in Table 2, and not in connection with a frequency count. We have checked all instances of frequency counts of n=7 and n=13 and none of them appear to be to do phenomena that are synonymous with discomfort. We do not understand the other two questions.

Line 305-312 seem to be more explanatory than rough data presentation. Why in the results section? Are other explanation possible or even likely? What about 25% of participants denying tests or investigations (line 314); representative for the health care seekers?

Sometimes in qualitative research it is useful, and accepted practice, to triangulate to the published literature during the Results. However, we realise this is unsettling to some reviewers. In deference to the reviewer, we have moved these lines to Line 431 of the Discussion.

Line 347; is fecal testing unfamiliar/uncommon in the Sheffield area?

In the journal’s PDF version of our original submitted manuscript, Line 347 reads. “347 inadequate pain control, or translation problems. Four discussed being told or”. We are struggling to understand the context of the reviewer’s question. Faecal testing is relatively common and familiar in the Sheffield area. However, this paper seeks to show variation in practice, and unevenness in its application.

**Discussion:**

The Safer-Andersen and Pedersen models are relevant for insiders, but a short introduction night be helpful, as holds true for psychophysiological comparison. It might be better to address why these models have been applied.

We have inserted the following, where these models are first introduced: “Psychophysiological Comparison Theory predicts that, when people notice symptoms, they try to understand their cause. They mentally compare the symptoms to their existing knowledge about illnesses. However, people are more likely to favour explanations that are less threatening. People can also describe their symptoms inaccurately because they have different perspectives and viewpoints when translating their subjective experiences into words. Social influences, especially wanting to meet certain expectations, can further change how people describe their symptoms. The Diagnostic Triage Model shows how people journey from first noticing symptoms to getting a diagnosis. There are decision points along the way - by you, your regular doctor, and specialist doctors. At each point, judgments are made – by people with symptoms, by generalists, by specialists, about how urgently the person need care. The model maps out the ups and downs on the path to diagnosis. Symptoms, people’s instincts, who they talk to, and interactions with doctors all impact the speed of progress. Delays happen when concerns are minimised; faster diagnosis comes through appropriate worry and referrals. The model aims to explain why some diagnosis journeys are smooth and some frustratingly prolonged. We chose these theories because they have evidential and coherential virtues[30]: they have shown good fit with the empirical evidence across a range of settings; and, they posit factors that plausibly cause the effect (diagnostic delay) in question which are coordinated in an intuitively plausible whole. It is appropriate to combine two theories or models to overcome the inherent limitation of each[31] - Psychophysiological Comparison Theory focusing on the patient and the Diagnostic Treatment Model on the wider system.”

Line 389 Is this a cognitive, a conative or an affective interpretation of the participant’s interview?

Line 389 in the journal’s PDF reads: "389 Change in the diagnosis prototype was changed by individuals after research - particularly on-line." The sentence appears in a discussion section and – as the use of the plural shows – relates to a generalisation based on several interviews, not that of a single participant. Our understanding is that this cognitive-conative-affective triad relates to German faculty psychology and the association psychologist, and that it is less in use since the early Twentieth Century (Hildard ER, Journal of the History of the Behavioral Sciences 16 (1980):107-117). We do not understand Psychophysiological Comparison Theory as consistent with the triad – it is a modern cognitive-affective theory, with no explicit reference to conation, and much more emphasis on cognition. For these reasons we do not feel comfortable applying synoptic/categorical theories to our data in the way the reviewer requests.
