# Supplementary material for: Sources of diagnostic delay for people with Crohn’s disease and ulcerative colitis: Qualitative research study
Source: PLoS One. 2024 Jun 10;19(6):e0301672. doi: 10.1371/journal.pone.0301672 (PMC11164383; doi:10.1371/journal.pone.0301672)
Supplement: Attachment — Submitted filename: Response to reviewers.docx [file pone.0301672.s003.docx]

**Response to reviewers**

Thank you for the helpful and constructive comments regarding the resubmission of this manuscript. We have responded to the reviewer comments below:

1. The authors question what was meant by "Expression of discomfort was present in 7 out of 20 participants?.....non-IBD 'abdominal' patients? ".The questions were mentioned to address line 295. 'Thirteen patients expressed comfort in discussing symptoms', leaving potentially 13 with discomfort. Or had they/these 13 no particular mention of any feeling? The third question was asked to clarify whether this 'discussion on symptoms' was (felt to be) socially embarrassing (in general whilst having abdominal complaints or was it a specific feature of IBD patients only?).

We have added the following sentence to clarify expressions of comfort and discomfort amongst the twenty participants – lines 284 – 286. “Of the remaining seven participants, six expressed some discomfort in discussing symptoms during their consultations and one could not recall how they felt.”

Although we want to clarify that these were qualitative interviews involving open questions, so participants were not restricted to comfort or discomfort as responses.

Regarding whether embarrassment during discussion of symptoms is a specific feature of IBD patients only, all twenty participants had some form of Crohn’s disease or ulcerative colitis. Interview questions focused on participant’s experiences discussing symptoms before their diagnosis, which often took the form of abdominal complaints. Therefore, we cannot say whether embarrassment when discussing symptoms is a specific feature of IBD patients only.

2. The second enigmatic question concerned fecal testing (apparently I made a typo with the correct number of the particular line). In my country GP-initiated (laboratory) diagnostic work-up of abdominal complaints includes fecal calprotectine assessment, being an important step for referring for colonoscopy at short notice (or a red flag for rapid / prioritized specialist consultation). In Table 3 only 6 check marks are indicated for 'Elevated calprotectin', making it difficult to interpret its meaning in standard Welsh (Sheffield) practice. Hopefully, with this, the context of these particular questions is clarified.

Calprotectin is now a part of the routine work-up of suspected inflammatory bowel disease with general practitioners following local specialist guidance. This paper seeks to show patient reported variation in practice, and unevenness in its application. Clinicians in our study did not have full access to GP notes.

Journal requirement:

Please review your reference list to ensure that it is complete and correct. If you have cited papers that have been retracted, please include the rationale for doing so in the manuscript text, or remove these references and replace them with relevant current references. Any changes to the reference list should be mentioned in the rebuttal letter that accompanies your revised manuscript. If you need to cite a retracted article, indicate the article’s retracted status in the References list and also include a citation and full reference for the retraction notice.

References list and citations have been corrected.
